# Clinical Outcomes and Treatment Strategies of Adult Transplant‐Associated Thrombotic Microangiopathy: External Validation of Harmonizing Definitions and High‐Risk Criteria

**DOI:** 10.1002/ajh.27651

**Published:** 2025-03-06

**Authors:** Aldo A. Acosta‐Medina, Meera Sridharan, Ronald S. Go, Ann M. Moyer, Nelson Leung, Maria Alice V. Willrich, Robert Wolf, Rabee Kassis, Almothana Manasrah, Mira A. Kohorst, Urshila Durani, Aasiya Matin, Mehrdad Hefazi, Saad J. Kenderian, Abhishek A. Mangaonkar, Mithun V. Shah, Mark R. Litzow, William J. Hogan, David Dingli, Hassan B. Alkhateeb

**Affiliations:** ^1^ Division of Hematology Mayo Clinic Rochester Minnesota USA; ^2^ Department of Laboratory Medicine and Pathology Mayo Clinic Rochester Minnesota USA; ^3^ Division of Nephrology and Hypertension Mayo Clinic Rochester Minnesota USA; ^4^ Department of Pharmacy Services Mayo Clinic Rochester Minnesota USA; ^5^ Department of Pediatric and Adolescent Medicine Mayo Clinic Rochester Minnesota USA

**Keywords:** allogeneic transplantation, complement inhibitor, risk assessment, thrombotic microangiopathy

## Abstract

Transplant‐associated thrombotic microangiopathy (TA‐TMA) is an endothelial dysfunction syndrome observed after allogeneic hematopoietic cell transplant (alloHCT). Our aim was to externally validate the impact of high‐risk features on the clinical outcomes of adult patients meeting the updated TA‐TMA harmonizing criteria. Between 2005 and 2022, 99 patients were diagnosed with TA‐TMA at Mayo Clinic Rochester (incidence 6.2%) after a median of 137 days post alloHCT (IQR: 34–283 days). The development of TA‐TMA was associated with an inferior overall survival posttransplant (HR: 3.8, 95% CI: 2.97–4.72). High‐risk features, including concomitant infection, acute graft‐versus‐host disease (GVHD), and organ dysfunction, were associated with poor survival, while LDH elevation was not associated with inferior outcomes. The most common treatment strategy for TA‐TMA was discontinuation of calcineurin or mTOR inhibitors in 80 (81%) patients. Thirty (37.5%) patients experienced worsening of GVHD with this strategy, of which 26 (86.7%) patients had died at last follow‐up. The most common cause of death among these patients was worsening GVHD (69%; *n* = 18), followed by infection (11%; *n* = 3), disease relapse (8%; *n* = 2), other/unknown causes (8%; *n* = 2), or TA‐TMA (4%; *n* = 1). Objective response rate (ORR) to initial treatment for the cohort was 56.6%. Eculizumab was used in 11 patients with an observed ORR of 70%, including 5 complete responses. In conclusion, TA‐TMA remains a significant contributor to non‐relapse mortality and is associated with worse survival following alloHCT. Not all high‐risk features, particularly LDH elevation, have consistently demonstrated a negative impact in adult cohorts. Patients with TA‐TMA may benefit from immune suppression dose adjustment, rather than a discontinuation, and the addition of complement‐directed therapy, particularly among high‐risk patients.

## 1 Introduction

Transplant‐associated thrombotic microangiopathy (TA‐TMA) is a rare endothelial dysfunction syndrome observed primarily after allogeneic hematopoietic stem cell transplant (alloHCT) and is associated with a high mortality [1, 2]. Though its pathogenesis remains an area of active study, complement activation—due to a variety of insults in genetically susceptible individuals—is thought to converge into diffuse proinflammatory signaling and consequent organ dysfunction in a similar fashion to that observed in other microangiopathic processes [3, 4, 5, 6].

There have been multiple efforts to homogenize the diagnostic criteria for TA‐TMA and to assess prognostic features associated with non‐relapse mortality (NRM) [7, 8, 9, 10, 11, 12], and to guide the use of therapeutic strategies, including terminal complement inhibition. Most recently, the harmonizing criteria from an international panel generated consensus statements regarding diagnostic and prognostic features of TA‐TMA [13] and a prospective, single‐arm, multi‐institutional trial on pediatric patients demonstrated improvement in clinical outcomes of high‐risk TA‐TMA with the use of eculizumab, a C5‐directed complement inhibitor, when compared to historical cohorts [14].

While the majority of data pertaining to TA‐TMA has been drawn from the pediatric experience, it remains uncertain whether all high‐risk prognostic features outlined in the harmonized consensus [13] carry the same effect in adult populations as they do in pediatric cohorts, particularly outside of the young adult age range. Herein, we present the clinical characteristics and outcomes of adult TA‐TMA as well as the use of complement inhibition in this population.

## 2 Methods

We performed a retrospective review of all consecutive adults undergoing alloHCT at Mayo Clinic Rochester between 2005 and 2022 to assess for clinical features suggestive of TA‐TMA. Patients were included upon diagnosis of TA‐TMA as per harmonized criteria and high‐risk features at the time of diagnosis as per published definitions [13]. Features for high‐risk TA‐TMA included: (i) a random urinary protein‐to‐creatinine ratio (rUPCR) ≥ 1 mg/mg, (ii) soluble C5b‐9 complex (sC5b‐9) plasma levels ≥ 250 ng/mL as per our institution's laboratory upper limit of normal (ULN), (iii) peak lactate dehydrogenase (LDH) ≥ 2 times the ULN, (iv) concurrent Grade ≥ 2 acute graft‐versus‐host disease (GVHD), (v) concurrent infection, and (vi) development of end‐organ dysfunction. Random UPCR evaluation was not routinely available and assessed in the alloHCT population until October 2010 and, likewise, the first evaluation of sC5b9 plasma levels in our cohort was in January 2017. Organ dysfunction considered for the definition of high‐risk disease included [14]: ≥ 50% reduction in glomerular filtration rate as compared to pretransplant value, any need for positive‐pressure ventilation for ≥ 24 h in the absence of clear etiology, diffuse alveolar hemorrhage, pulmonary hypertension diagnosed by a cardiologist either through right cardiac catheterization or echocardiography, pleural/pericardial effusion or ascites requiring medical therapy or drainage in the absence of a clear etiology, seizures and/or imaging evidence of posterior reversible encephalopathy syndrome, altered mental status without clear etiology after comprehensive evaluation by a neurologist, and gastrointestinal bleeding in the absence of a clear etiology.

Patients were considered to have received eculizumab therapy if ≥ 1 dose was administered for attempted disease management. Dosing of eculizumab in the cohort was based on the drug label at weekly intervals for the first four doses.

Objective response rate (ORR) was defined as patients achieving a complete response (CR) or partial response (PR) after therapy initiation. A CR was defined as the resolution of hemolysis parameters, the absence of ongoing transfusion needs—unless otherwise explained by a demonstrated, separate etiology—, and the return of renal function and proteinuria to baseline. Patients were classified as not having achieved a response (NR) if they did not achieve the resolution of hemolysis and the return of renal function and proteinuria to baseline. All other patients were considered to have a PR. GVHD staging was defined per international standards for acute GVHD [15] and chronic GVHD [16], respectively.

Demographic, laboratory, and clinical data were abstracted from electronic medical records. The study was deemed exempt by our Institutional Review Board and was performed in accordance with the Declaration of Helsinki.

### 2.1 Statistical Analysis

Nominal and ordinal variables were described in terms of frequency and percentage, while quantitative data were described in terms of median and range or interquartile range. The statistical comparison of categorical variables was performed using the chi‐square test, and the *t* test was used for the comparison of continuous variables.

Median follow‐up was determined using the reverse Kaplan–Meier method. To assess the effect of TA‐TMA on overall survival (OS) across all patients who underwent alloHCT in the study period, the development of TA‐TMA was incorporated as a time‐dependent covariate and tested via standard Cox regression assays [17]. Subsequent survival analyses were restricted to the TA‐TMA patient population. Time‐to‐event survival curves were constructed via the standard Kaplan–Meier method and initially compared via the log‐rank test. The cumulative incidence of NRM was determined using competing risk analyses, with relapse considered a competing risk. Gray's analysis was used to compare differences between cumulative incidence curves [18]. A standard Cox regression was performed for the creation of the multivariable OS analysis, while a Fine‐Gray analysis was used for the multivariable NRM competing risk regression analysis. Given the preestablished evidence for significant effects of all high‐risk features on survival outcomes in the available literature, all high‐risk features were included in the multivariable models constructed, regardless of statistical significance observed in univariate testing.

All statistical analysis was performed using R, version 4.2.0 (R foundation for statistical computing, Vienna, Austria); the level of significance was set at *p* < 0.05.

### 2.2 Results

Among a total 1574 patients undergoing alloHCT, 99 patients met the criteria for TA‐TMA (6.3%) with a median age of 56 years (IQR: 47–61 years) at the time of transplantation. Patients with TA‐TMA were more commonly female (52/99, 52.5%) and the most common indications for transplantation were acute myeloid leukemia (43/99, 43%) and myelodysplastic syndromes (13/99, 13%). Regarding alloHCT characteristics, patients most commonly received a T‐cell replete peripheral blood stem cell graft (93/99, 94%) from a matched unrelated (45/99, 46%) or related (43/99, 43%) donor after a reduced intensity conditioning regimen (58/99, 59%). GVHD prophylaxis included a calcineurin inhibitor (CNI) in combination with methotrexate or mTOR pathway inhibitor (mTORi) in 98% and 12% of the patients, respectively. Additional detailed baseline characteristics are presented in Table 1.

**TABLE 1:** Baseline patient characteristics.

Transplant characteristics	*N* = 99	High‐risk features^d^	*N* = 99
Age at alloSCT, median (IQR)	56 (47–61)	Lactate dehydrogenase, *n* (%)	
		*x*2 < ULN	24 (24.2)
		*x*2 ≥ ULN	75 (75.8)
Female sex, *n* (%)	52 (53)	Concomitant infection, *n* (%)	
		No	45 (45.5)
		Yes	54 (54.5)
Race, *n* (%)		Acute graft‐versus host disease, *n* (%)	
White	91 (91.9)	< Grade 2	55 (55.6)
Other	8 (8.1)	≥ Grade 2	44 (44.4)
Primary hematologic diagnosis, *n* (%)		End‐organ dysfunction, *n* (%)	
Acute myeloid leukemia	43 (43.4)	Present	35 (35.4)
Myelodysplastic syndrome	13 (13.1)	Pulmonary	18
Acute lymphoblastic leukemia	13 (13.1)	Central nervous system	16
Multiple myeloma	11 (11.1)	Serositis	3
Chronic leukemia^a^	8 (8.1)	Cardiovascular	1
Lymphoma^b^	6 (6.1)		
Other^c^	5 (5)		
Graft type, *n* (%)		Proteinuria, *n* (%)	
Peripheral blood	93 (94)	Urine protein‐to‐creatinin ratio < 1	34 (34.3)
Bone marrow	3 (3)	Urine protein‐to‐creatinin ratio ≥ 1	15 (15.2)
Cord	3 (3)	Not assessable	50 (50.5)
Donor type, *n* (%)		Complement activation, *n* (%)	
Matched related	43 (43.4)	Soluble C5b9 < ULN	10 (10.1)
Matched unrelated	46 (46.5)	Soluble C5b9 ≥ ULN	15 (15.2)
Haploidentical	7 (7.1)	Not assessable	74 (74.7)
Cord	3 (3)		
Conditioning intensity, *n* (%)			
Myeloablative	40 (40.4)		
Reduced‐intensity	58 (58.6)		
Non‐myeloablative	1 (1)		
Conditioning regimen, *n* (%)			
Fludarabine‐containing	66 (66.7)		
Melphalan‐containing	60 (60.6)		
Total body irradiation‐containing	34 (34.3)		
Cychlophosphamide‐containing	32 (32.3)		
Busulfan‐containing	14 (14.1)		
GVHD regimen, *n* (%)			
CNI‐containing	97 (98)		
mTOR inhibitor‐containing	12 (12.1)		
PTCy‐containing	7 (7.1)		

Abbreviations: alloHCT, allogeneic transplant; CNI, calcineurin inhibitor (tacrolimus/cyclosporine A); GVHD, graft‐versus‐host disease; mTOR, mechanistic target of rapamycin (sirolimus); PTCy, posttransplant cyclophosphamide.

^a^ Myelomonocytic (*n* = 4), myeloid (*n* = 3), and lymphoid (*n* = 1).

^b^ B‐cell (*n* = 2), T‐cell (*n* = 2), and Hodgkin (*n* = 2).

^c^ Primary myelofibrosis (*n* = 2), plasmacytoid dendritic cell neoplasm (*n* = 2), and Aplastic anemia (*n* = 1).

^d^ Jodele et al. [14].

Diagnosis of TA‐TMA occurred at a median of 137 days post alloHCT (IQR: 34–283 days) with 47% of cases diagnosed before Day +100. Overall, 16% of TA‐TMA had biopsy‐proven TA‐TMA, and all non‐biopsy proven cases met clinical criteria for TA‐TMA [13]. The median time from first clinical or laboratory features suggestive of TA‐TMA to diagnosis was 6 days (IQR: 2–16 days) with this time frame extending to ≥ 30 days in 15% of patients.

At the time of diagnosis of TA‐TMA, only 49 (49%) patients had the required urinary work‐up to determine whether high‐risk proteinuria was present. Likewise, complement sC5b‐9 activity was only evaluable in 25 (25%) of the more contemporary TA‐TMA cases. However, 98% of patients (*n* = 97) met criteria for high‐risk TA‐TMA based on harmonizing criteria. Detailed information pertaining to high‐risk features observed in the cohort is presented in Table 1. Among patients with a diagnosis of TA‐TMA beyond Day +100, 45/53 (85%) had either history of or active acute or chronic GVHD, while only eight patients (15%) did not have any prior or active GVHD. Among those with late TA‐TMA and a history of or active GVHD, 23/45 (51%) had ongoing late onset or prior acute GVHD, while 15/45 (33.3%) had evidence of active chronic GVHD at the time of TA‐TMA diagnosis, and 7/53 (15.5%) had a history of previously treated chronic GVHD.

#### 2.2.1 Survival Analysis

**FIGURE 1:**
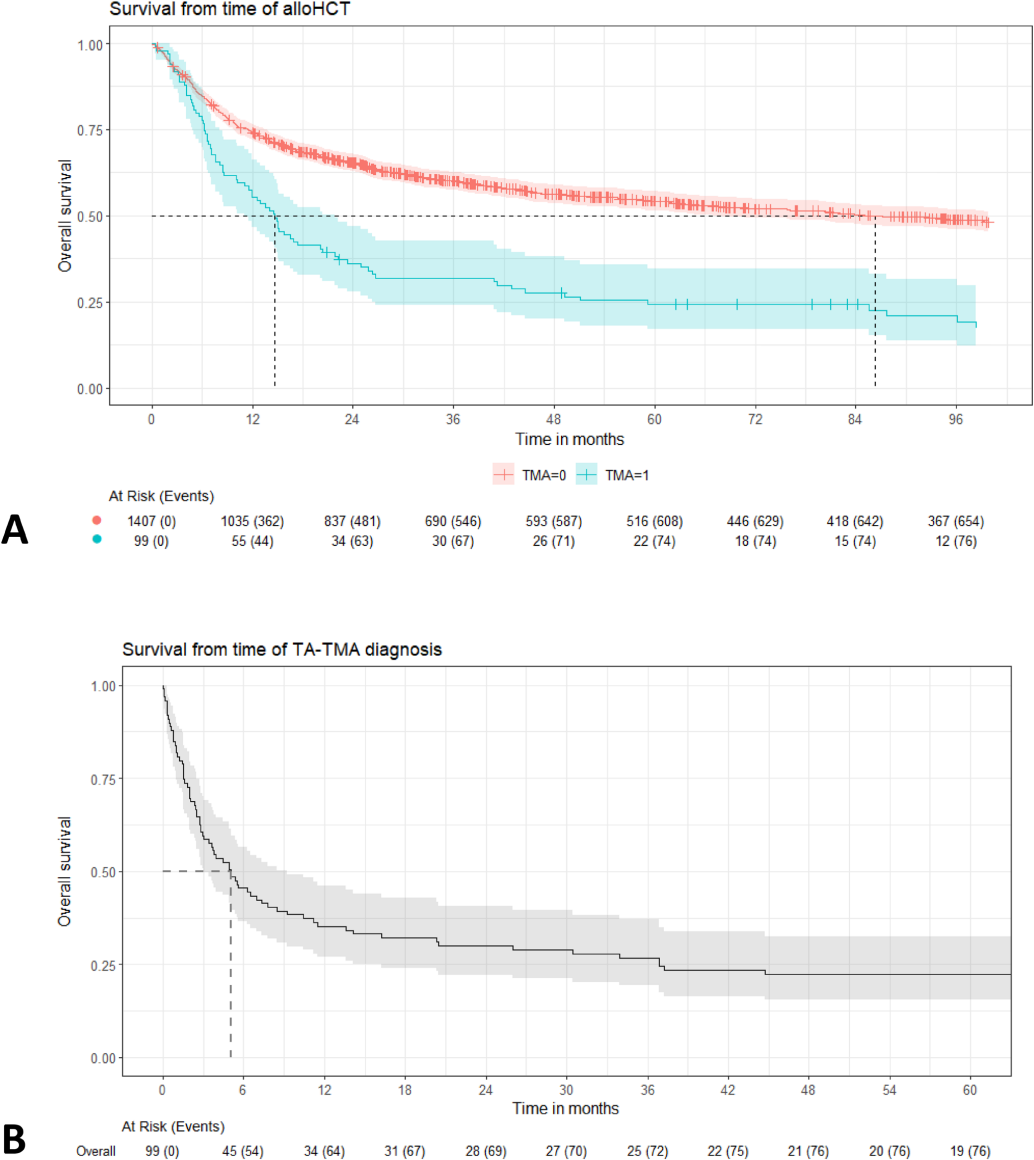
Overall survival for (A) all allogeneic transplant recipients from time of transplantation according to development of transplant‐associated thrombotic microangiopathy (TMA) and (B) from time of TMA diagnosis among the 99 patients diagnosed across the study period. [Color figure can be viewed at wileyonlinelibrary.com]

After a median follow‐up of 8.5 years for the whole cohort, the median OS from alloHCT was 6.3 years (95% CI: 4.9–7.9 years). Considering TA‐TMA as a time‐dependent variable, the development of TA‐TMA was associated with an inferior OS compared to the rest of the cohort (median 1.2 vs. 7.2 years; HR: 3.8, 95% CI: 2.97–4.72; *p* < 0.001; Figure 1A). Patients developing TA‐TMA had a median OS from diagnosis of 5 months (95% CI: 3–9 months; Figure 1B) with 81 deaths (82%) recorded at the time of last follow‐up.

**FIGURE 2:**
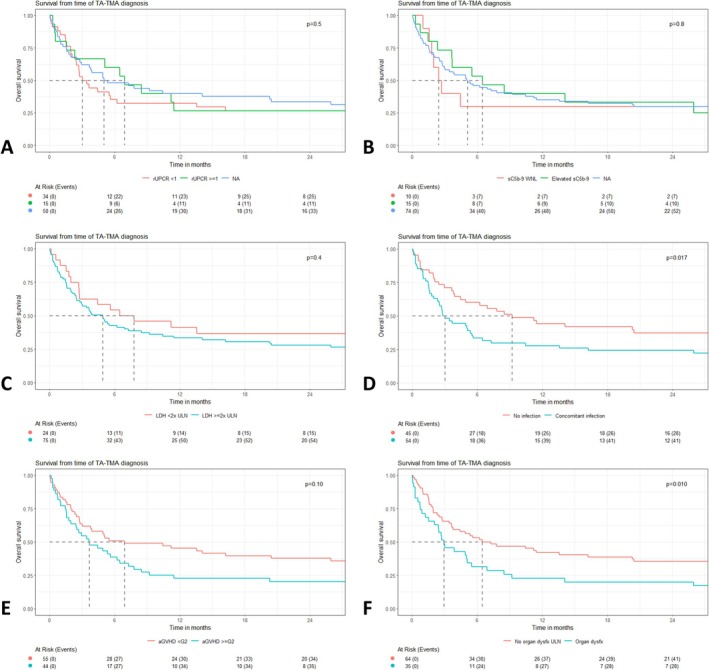
Univariate analysis of overall survival from time of TA‐TMA diagnosis according to high‐risk features including: (A) random urine protein‐to‐creatinine ratio (rUPCR), (B) soluble C5‐b9 levels (sC5b‐9), (C) lactate dehydrogenase (LDH) levels, (D) presence of concomitant infection, (E) concomitant Grade ≥ 2 acute graft‐versus‐host disease (aGVHD), or (F) presence of organ dysfunction. *p* Values expressed represent log‐rank testing. *Note*: 7 out of 15 patients included in the elevated sC5b‐9 curve received eculizumab. [Color figure can be viewed at wileyonlinelibrary.com]

Univariate analysis of high‐risk TA‐TMA features (Figure 2) demonstrated factors associated with worsened OS following TA‐TMA included: concomitant infection (*p* = 0.017), Grade ≥ 2 acute GVHD (*p* = 0.1), and organ dysfunction (*p* = 0.01). Multivariable analysis demonstrated significantly inferior OS among patients with organ dysfunction (HR: 2.26, 95% CI: 1.36–3.74; *p* = 0.002), concomitant infection (HR: 1.81, 95% CI: 1.15–2.85; *p* = 0.011), and acute GVHD (HR: 1.73, 95% CI: 1.08–2.78; *p* = 0.023).

**FIGURE 3:**
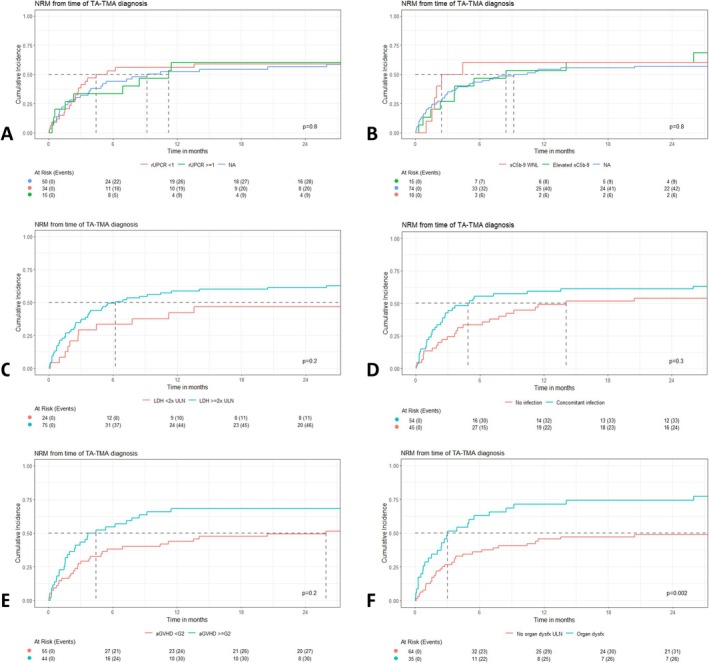
Univariate analysis of non‐relapse mortality (NRM) from time of TA‐TMA diagnosis according to high‐risk features including: (A) random urine protein‐to‐creatinine ratio (rUPCR), (B) soluble C5‐b9 levels (sC5b‐9), (C) lactate dehydrogenase (LDH) levels, (D) presence of concomitant infection, (E) concomitant Grade ≥ 2 acute graft‐versus‐host disease (aGVHD), or (F) presence of organ dysfunction. *p* Values expressed represent Gray's test. [Color figure can be viewed at wileyonlinelibrary.com]

On univariate analysis of high‐risk TA‐TMA features (Figure 3), NRM was significantly worsened in patients with organ dysfunction (*p* = 0.002) at the time of TA‐TMA diagnosis. However, multivariable analysis showed an increased cumulative hazard of NRM among patients with acute GVHD (HR: 1.73, 95% CI: 1.05–2.85; *p* = 0.032) and organ dysfunction (HR: 2.26, 95% CI: 1.31–3.89; *p* = 0.004).

The most common causes of death following TA‐TMA included GVHD (36%, *n* = 29), infectious complications (24%, *n* = 19), TA‐TMA (19%, *n* = 15), and primary disease relapse (12%, *n* = 10). Given the excess representation of non‐TMA‐specific causes of death and the lack of clear evidence of negative clinical impact of high‐risk TA‐TMA features on survival outcomes, additional exploratory analyses were pursued.

### 2.3 Treatment Strategies in Adult TA‐TMA

ORR to initial treatment for the cohort was 56.6%. The most common initial treatment strategy after the diagnosis of TMA was the discontinuation of CNI or mTORi, which accounted for 81% (*n* = 80) of patients. Among these, 32 (40%) patients had another concomitant treatment strategy initiated at the time of drug discontinuation, most frequently either therapeutic plasma exchange (PLEX; *n* = 21) or terminal complement inhibition via eculizumab (*n* = 5). Initiation of a non‐CNI/mTOR agent such as mycophenolate mofetil (MMF) was universal in the remaining patients unless GVHD prophylaxis tapering had already been initiated before TA‐TMA development. Other initial treatment for TA‐TMA included CNI/mTORi dose reduction (*n* = 8, 8%), PLEX and/or rituximab (*n* = 7, 7%), initiation of other immunosuppressive agents (*n* = 3, 3%; e.g., tocilizumab, high‐dose steroids), or treatment of presumed triggering infection (*n* = 1, 1%).

A total of 30 (30%) patients experienced worsening of GVHD after undergoing the first therapeutic intervention for TA‐TMA. All of whom had CNI/mTORi discontinuation, and 26 (86.7%) had died at the last follow‐up. The most common cause of death among these patients was GVHD in 69% (*n* = 18), followed by infectious complications in 11% (*n* = 3), relapse in 8% (*n* = 2), other/unknown in 8% (*n* = 2), or TA‐TMA in 4% (*n* = 1).

### 2.4 Eculizumab‐Receiving Cohort

A total of 11 patients received eculizumab as part of their therapy for TA‐TMA. Additional features pertaining to baseline characteristics and clinical course of these patients are presented in Table S1. Among patients with available sC5b‐9, there was a significant association between elevated sC5b‐9 levels and administration of eculizumab (*p* < 0.001). Diagnosis of TA‐TMA in this subgroup occurred after a median of 86 days post alloHCT (IQR: 23–181). All 11 patients met criteria for high‐risk TA‐TMA based on current harmonized criteria [13]. Specific high‐risk criteria included: proteinuria in 2/6 (30%; 5 patients without necessary urinary testing), elevated sC5b‐9 in 7/9 (78%; median 347 ng/mL, IQR: 246–459; 2 patients without necessary testing), LDH elevation in 11/11 (100%), concomitant infection in 6/11 (55%), acute GVHD in 4/11 (36%), and end‐organ dysfunction in 5/11 (45%). Diagnosis of TA‐TMA was established within a median of 6 days (IQR: 4–17) from the first sign of hemolysis or TMA‐associated end‐organ dysfunction.

Initial management after diagnostic confirmation of TA‐TMA included CNI/mTORi discontinuation in all but one patient (82%). Patient #18 continued therapy with CNI and was initially treated with PLEX until the initiation of eculizumab 16 days after the first signs of TMA due to a lack of response. Overall, five patients had an intent to initiate eculizumab as part of first‐line therapy, while the remaining six patients initiated eculizumab at the discretion of the treating physician after a lack of clear clinical improvement with the first treatment line, generally within the first 2 weeks of the first intervention.

Among patients with evaluable response, the ORR was 70% with a median eculizumab dose of 4 (range 1–12 doses). One patient transitioned to hospice shortly after the first eculizumab dose was administered, and the response was not assessable, while a CR and a PR were achieved in 5/10 and 2/10 patients, respectively.

Initial improvement in platelet counts and hemolysis parameters was noticed within the first week after infusion in most patients with a response, particularly in those achieving a CR, with a median time to hematologic response of 16 days (range 8–37 days; Table S1) and a median time to best response of 35 days (range 19–63 days) from the first eculizumab infusion. There was no clear association between the time of initiation of eculizumab and the best response achieved. Both patients achieving a PR initiated eculizumab more than 1 week after TA‐TMA diagnosis, and a CR was observed in a patient initiating therapy after 20 days from diagnosis, while the three patients without response had initiated eculizumab within 7 days of diagnosis.

Among patients who achieved a PR, one experienced a full hematologic response with partial renal recovery and new KDIGO Stage III chronic kidney disease (Patient #7) and one had a partial hematologic response without renal improvement (Patient #98).

At last follow‐up, none of the responding patients experienced TA‐TMA relapse. Notably, 4/10 patients, of whom GVHD prophylaxis was discontinued for TA‐TMA, had worsening acute or chronic GVHD. Within this subgroup, two patients developed Grade IV gastrointestinal GVHD. Patient #4's course was complicated by rectal perforation demonstrating polymicrobial growth, including *Candida glabrata* , and Patient #101 became steroid‐dependent and developed diverticulitis with concomitant candidemia. No additional invasive fungal infections were identified in the eculizumab‐receiving cohort.

At the time of the last follow‐up, eight deaths were recorded, including: all nonresponding patients (Patients #1, #4, #18, and #96), two patients with infectious complications more than 2 years following TA‐TMA (Patients #12 and #145), and two patients who elected to transition to comfort care due to complications from GVHD despite achieving CR (Patient #101) while the other had TMA‐related renal dysfunction (Patient #98).

### 2.5 Discussion

Our study represents the first adult cohort to validate the effect of high‐risk TA‐TMA features on the clinical outcomes of patients meeting updated harmonizing criteria [13] and one of the largest cohorts of adult patients developing TA‐TMA following alloHCT in the published literature [12, 19, 20].

There was an observed incidence of TA‐TMA of 6% across 17 years within our center. While contemporary pediatric cohorts have reported rates in the 15%–25% [21, 22], this low incidence is in line with that reported in the adult literature [23]. A clear cause for these stark age‐related differences has not been identified. However, impacting factors may encompass differences between pediatric and adult transplant practices, including those in conditioning regimen intensity (i.e., increased use of reduced‐intensity conditioning) and use of specific conditioning agents (e.g., negligible use of high‐dose carboplatin/etoposide/melphalan), as well as the overall effects of cellular senescence on the ability to mount a brisk immune response [24]. The latter may also aid in explaining the relatively young patient population (median age 51 years) seen in both our cohort and large TA‐TMA reports [12] despite progressive increases in upper age limits for alloHCT in the past decades [25].

In terms of findings at the time of diagnosis, the most frequent signs triggering additional TMA evaluation included the development of acute kidney injury and/or the report of schistocytes on a peripheral smear. Most patients without clear evidence of a vigorous microangiopathic process and whose diagnosis relied on tissue biopsy were diagnosed in the post‐2015 era, suggestive of increased physician awareness of the broad spectrum of features of TA‐TMA. Additionally, as per our group's previously published experience [26], adult TA‐TMA should not only be considered an early complication following alloHCT, as evidenced by half our cohort fulfilling diagnostic criteria after the “traditional” Day +100 cutoff. Strikingly, most patients (85%) presenting with late TA‐TMA had either a prior incident or active GVHD, while only 15% of patients did not experience any GVHD. In late TA‐TMA, there is a high association between the incidence of GVHD and microangiopathy, while the frequency of having a history of or active acute or chronic GVHD was almost identical.

Notably, a large proportion of patients, particularly, those with less recent diagnoses, did not have the required terminal complement activation or proteinuria data to assess for features of high‐risk TA‐TMA. Despite this, high‐risk TA‐TMA was nearly universal.

Further review of the impact of the six currently defining features of high‐risk disease in our cohort only demonstrated evidence of a deleterious effect on mortality outcomes in cases with concomitant infections, acute GVHD, and end‐organ dysfunction at the time of TA‐TMA diagnosis. Lack of a discernible negative effect of proteinuria and an elevated sC5b‐9 at diagnosis could be partly attributed to two factors: (i) the reduced sample size with assessable parameters and (ii) the varying treatment strategies for TA‐TMA throughout the study period with an evident bias toward treatment with eculizumab in patients with known elevated complement activation parameters (7/15, 47%). In contrast, these do not hold true for the use of an elevated LDH as a prognostic feature. Importantly, most data linking an LDH ≥ 2 times the ULN with poor prognosis in TA‐TMA is derived from the pediatric literature [27, 28] or young adults in whom LDH was used as a defining characteristic of microangiopathy [29]. Additionally, an elevated LDH was a feature present in more than 75% of our cohort further limiting the discriminatory capacity of the test into clinically meaningful subgroups. Our results argue against the use of LDH for discrimination of high‐risk TA‐TMA in the adult population.

While the heterogeneity and sample size of non‐CNI/mTORi discontinuation strategies limited our ability for direct statistical comparisons between treatment subgroups, our results demonstrate the nuanced nature of immune suppression management in alloHCT recipients. Though the only additional US‐based cohort to report clinical outcomes of immune suppression withdrawal in TA‐TMA suggested similar mortality outcomes between the drug withdrawal and non‐withdrawal subgroups [12], our results highlight the increased mortality with discontinuation or switch of CNIs that was previously observed by the Kyoto Stem Cell Transplantation Group [19]. In our cohort, GVHD was the most common cause of death. However, more than half of the GVHD‐related deaths (18/29, 62%) were directly attributable to worsening acute GVHD following CNI/mTOR discontinuation.

Response to eculizumab was heterogeneous, although it appeared improved when compared to historical cohorts, particularly, when considering that all patients met criteria for high‐risk TA‐TMA [30, 31]. There were no cases of meningococcal bacteremia or invasive mold infections. While there were two cases of invasive candidiasis, both patients had severe acute gastrointestinal GVHD flare following CNI/mTOR discontinuation, in line with previous pediatric reports [32]. Importantly, only two deaths were reported in the first 2 years following TA‐TMA in patients responding to eculizumab (2/7, 29%), only one of which was attributable to TMA. Though limited samples restricted statistical testing, there was a clear trend toward increased sC5b‐9 levels at TA‐TMA diagnosis in patients who achieved a CR with eculizumab (median 971 vs. 343 ng/dL if≤PR) that should be further explored in the future.

Taking all the previous data into consideration, our group proposes and has adopted a therapeutic approach toward the management of recently diagnosed adult TA‐TMA (Figure S1) which emphasizes adjustment of CNI/mTOR dosing exclusively in patients in whom supratherapeutic drug levels have been confirmed and are thought to be possible contributors to TA‐TMA development. If terminal complement blockade is initiated, standard practice should include the initiation of appropriate antimicrobial prophylaxis with meningococcal coverage given the anticipated decrease in efficacy in active immunization in the alloHCT population.

The main limitations of our study include its retrospective nature leading to possible underestimation of the true incidence rates of adult TA‐TMA, restricted racial representation with more than 90% of the cohort being non‐Hispanic Caucasians, as well as the absence of urinary and complement activity parameters in a large proportion of the cohort. We are reassured that underestimation may be limited given the intergroup consistency when compared with other adult referral centers [12]. However, it is possible that patients with subclinical, low‐risk TA‐TMA were not being identified, particularly, past the first 100 days following transplantation when laboratory assessments are typically done less frequently. Germline genetic testing and pharmacokinetics were not available among the eculizumab‐receiving group.

In conclusion, TA‐TMA is rare but remains a significant contributor to NRM in adults undergoing alloHCT. These patients may benefit from immune suppression dose adjustment and the addition of complement‐directed therapy, specifically among non‐responders to initial strategies or with evidence of high‐risk TA‐TMA at diagnosis. Lack of response to these strategies remains a challenge and often leads to rapid decline and death. The use of baseline and at‐time‐of‐diagnosis sC5b‐9 levels to guide intensification of therapy or likelihood of response to eculizumab remains an area in need of further exploration. The study of additional therapeutic options and joint multi‐institutional efforts will be pivotal in this population.

## Author Contributions

**Aldo A. Acosta‐Medina:** data curation, methodology, investigation, writing the initial draft. **Meera Sridharan:** data and patient contribution, review and approval of manuscript. **Ronald S. Go:** data and patient contribution, review and approval of manuscript. **Ann M. Moyer:** resources, data curation/investigation, review and approval of manuscript. **Nelson Leung:** data and patient contribution, review and approval of manuscript. **Maria Alice V. Willrich:** resources, data curation/investigation, review and approval of manuscript. **Robert Wolf:** data contribution, review and approval of manuscript. **Rabee Kassis:** data curation. **Almothana Manasrah:** data curation. **Mira A. Kohorst:** data and patient contribution, review and approval of manuscript. **Urshila Durani:** data and patient contribution, review and approval of manuscript. **Aasiya Matin:** data and patient contribution, review and approval of manuscript. **Mehrdad Hefazi:** data and patient contribution, review and approval of manuscript. **Saad J. Kenderian:** data and patient contribution, review and approval of manuscript. **Abhishek A. Mangaonkar:** data and patient contribution, review and approval of manuscript. **Mithun V. Shah:** data and patient contribution, review and approval of manuscript. **Mark R. Litzow:** data and patient contribution, review and approval of manuscript. **William J. Hogan:** data and patient contribution, review and approval of manuscript. **David Dingli:** data and patient contribution, review and approval of manuscript. **Hassan B. Alkhateeb:** conceptualization, methodology, supervision, reviewed the initial draft, and reviewed and approved the final draft.

## Ethics Statement

The study was deemed exempt by our Institutional Review Board and performed in accordance with the Declaration of Helsinki.

## Conflicts of Interest

M.V.S.: Research funding to the institution by AbbVie, Astellas, Celgene, KURA, and MRKR Therapeutics. M.R.L.: Research funding by Actinium, Amgen, Abbvie, Astellas, Pluristem, Sanofi; Speaker's Bureau at Amgen, Beigene; and Data Safety Monitoring Committee for Biosight. The other authors declare no conflicts of interest.

## Supporting information


**Figure S1.** Proposed treatment algorithm for management of newly diagnosed adult transplant‐associated thrombotic microangiopathy. CBC, complete blood count; CNI, calcineurin inhibitor; hr‐TMA, high‐risk transplant‐associated thrombotic microangiopathy; LDH, lactate dehydrogenase; mTORi, mammalian target of rapamycin inhibitor.

**Table S1.** Detailed baseline and clinical outcomes of patients receiving eculizumab.

## Data Availability

Data will be provided upon direct request to the authors. For original data request contact AcostaMedina.Aldo@mayo.edu and Alkhateeb.Hassan@mayo.edu.
