# Supplementary figures and images for: Clinical Outcomes and Treatment Strategies of Adult Transplant‐Associated Thrombotic Microangiopathy: External Validation of Harmonizing Definitions and High‐Risk Criteria
Source: Am J Hematol. 2025 Mar 6;100(5):830–9. doi: 10.1002/ajh.27651 (PMC11966343; doi:10.1002/ajh.27651)

## Slide 1
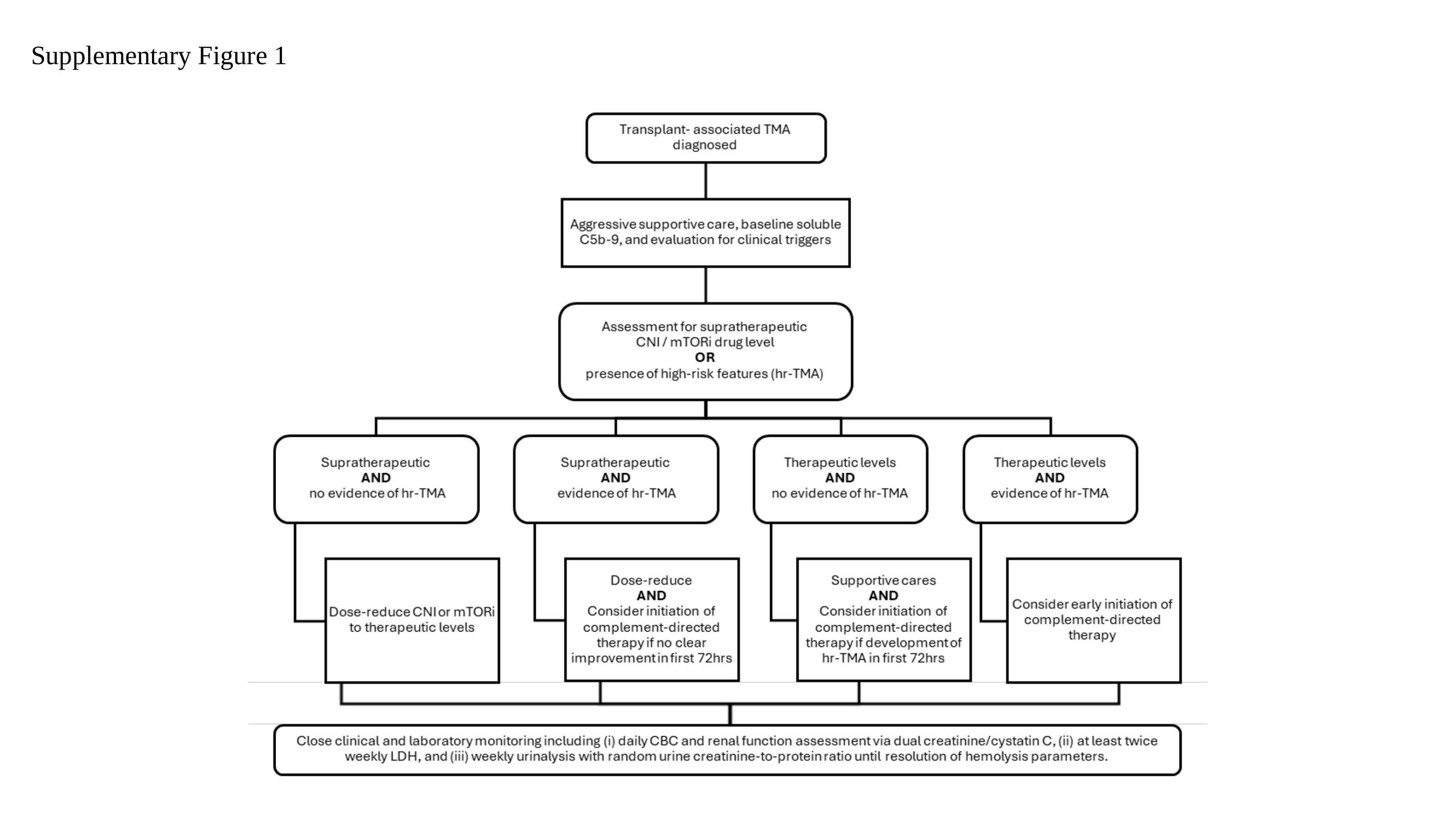

# Figure 1
Supplementary Figure 1

Supplement: Supplementary file 1 — Figure S1. Proposed treatment algorithm for management of newly diagnosed adult transplant‐associated thrombotic microangiopathy. CBC, complete blood count; CNI, calcineurin inhibitor; hr‐TMA, high‐risk transplant‐associated thrombotic microangiopathy; LDH, lactate dehydrogenase; mTORi, mammalian target of rapamycin inhibitor. [file AJH-100-830-s002.pptx]
