# Supplementary material for: Clinical Outcomes and Treatment Strategies of Adult Transplant‐Associated Thrombotic Microangiopathy: External Validation of Harmonizing Definitions and High‐Risk Criteria
Source: Am J Hematol. 2025 Mar 6;100(5):830–9. doi: 10.1002/ajh.27651 (PMC11966343; doi:10.1002/ajh.27651)
Supplement: Supplementary file 2 — Table S1. Detailed baseline and clinical outcomes of patients receiving eculizumab. [file AJH-100-830-s001.docx]

**Table and Figure Legends**

**Table 1:** Baseline patient characteristics

**Figure 1:** Overall survival for A) all allogeneic transplant recipients from time of transplantation according to development of transplant-associated thrombotic microangiopathy (TMA) and B) from time of TMA diagnosis among the 99 patients diagnosed across the study period.

**Figure 2:** Univariate analysis of overall survival from time of TA-TMA diagnosis according to high-risk features including: A) random urine protein-to-creatinine ratio [rUPCR], B) soluble C5-b9 levels [sC5b-9]*, C) lactate dehydrogenase [LDH] levels, D) presence of concomitant infection, E) concomitant grade ≥2 acute graft-versus-host disease [aGVHD], or F) presence of organ dysfunction. P values expressed represent log-rank testing.

* Note: 7 out of 15 patients included in the elevated sC5b-9 curve received eculizumab

**Figure 3:** Univariate analysis of non-relapse mortality [NRM] from time of TA-TMA diagnosis according to high-risk features including: A) random urine protein-to-creatinine ratio [rUPCR], B) soluble C5-b9 levels [sC5b-9], C) lactate dehydrogenase [LDH] levels, D) presence of concomitant infection, E) concomitant grade ≥2 acute graft-versus-host disease [aGVHD], or F) presence of organ dysfunction. P values expressed represent Gray’s test.

**Supplementary Table 1:** Detailed baseline and clinical outcomes of patients receiving eculizumab

**Supplementary Figure 1:** Proposed treatment algorithm for management of newly diagnosed adult transplant-associated thrombotic microangiopathy. CBC: complete blood count, CNI: calcineurin inhibitor, LDH: lactate dehydrogenase, mTORi: mammalian target of rapamycin inhibitor, hr-TMA: high-risk transplant-associated thrombotic microangiopathy.

*Table 1.*

| Transplant characteristics | *N* = 99 | High-risk features^£^ | *N* = 99 |  |
| --- | --- | --- | --- | --- |
| Age at alloSCT, *median* (IQR) | 56 (47 - 61) | **Lactate dehydrogenase, *n* (%)**  x2 < ULN  x2 >= ULN | 24 (24.2)  75 (75.8) |  |
| Female sex, *n* (%) | 52 (53) | **Concomitant infection, *n* (%)**  No  Yes | 45 (45.5)  54 (54.5) |  |
| Race, *n* (%)  White  Other | 91 (91.9)  8 (8.1) | **Acute graft-versus host disease, *n* (%)**  < Grade 2  >= Grade 2 | 55 (55.6)  44 (44.4) |  |
| Primary hematologic diagnosis, *n* (%)  Acute myeloid leukemia  Myelodysplastic syndrome  Acute lymphoblastic leukemia  Multiple myeloma  Chronic leukemia*  Lymphoma^€^  Other^α^ | 43 (43.4)  13 (13.1)  13 (13.1)  11 (11.1)  8 (8.1)  6 (6.1)  5 (5) | **End-organ dysfunction, *n* (%)**  Present   - Pulmonary - Central nervous system - Serositis - Cardiovascular | 35 (35.4)   - 18 - 16 - 3 - 1 |  |
| Graft type, *n* (%)  Peripheral blood  Bone marrow  Cord | 93 (94)  3 (3)  3 (3) | **Proteinuria, *n* (%)**  Urine protein-to-creatinin ratio < 1  Urine protein-to-creatinin ratio >= 1  Not assessable | 34 (34.3)  15 (15.2)  50 (50.5) |  |
| Donor type, *n* (%)  Matched related  Matched unrelated  Haploidentical  Cord | 43 (43.4)  46 (46.5)  7 (7.1)  3 (3) | **Complement activation, *n* (%)**  Soluble C5b9 < ULN  Soluble C5b9 >= ULN  Not assessable | 10 (10.1)  15 (15.2)  74 (74.7) |  |
| Conditioning intensity, *n* (%)  Myeloablative  Reduced-intensity  Non-myeloablative | 40 (40.4)  58 (58.6)  1 (1) |  |  |  |
| Conditioning regimen, *n* (%)  Fludarabine-containing  Melphalan-containing  Total body irradiation-containing  Cychlophosphamide-containing  Busulfan-containing | 66 (66.7)  60 (60.6)  34 (34.3)  32 (32.3)  14 (14.1) |  |  |  |
| GVHD regimen*, n* (%)  CNI-containing  mTOR inhibitor-containing  PTCy-containing | 97 (98)  12 (12.1)  7 (7.1) |  |  |  |
| alloHCT: allogeneic transplant, CNI: calcineurin inhibitor (tacrolimus / cyclosporine A), mTOR: mechanistic target of rapamycin (sirolimus), GVHD: graft-versus-host disease, PTCy: post-transplant cyclophosphamide.  * Myelomonocytic (n=4), Myeloid (n=3), Lymphoid (n=1). € B-cell (n=2), T-cell (n=2), Hodgkin (n=2). α Primary myelofibrosis (n=2), Plasmacytoid dendritic cell neoplasm (n=2), Aplastic anemia (n=1)  *£ Jodele et al, Blood. 24;143(12):1112-1123* | | | | |

*Supplementary Table 1.*

| **ID** | **Hematologic**  **Diagnosis** | **DRI risk*** | **Conditioning** | **Donor** | **CNI / mTORi exposure** | **High-risk features^α^** | **Total Eculizumab Doses** | **Time to Eculizumab Initiation** | **Response achieved** | **Time to Hematologic Response** | **Fungal complications** | **Status at last follow up** |
| --- | --- | --- | --- | --- | --- | --- | --- | --- | --- | --- | --- | --- |
| **1** | MDS | Intermediate | Flu/Mel | Haplo | Y / N | LDH, Infection | 1 | <72 hrs | N/A^Ɛ^ | N/A | N/A | Dead |
| **4** | AML | Intermediate | Flu/Mel | MUD | Y / N | LDH | 7 | <72 hrs | NR | N/A | C. glabrata in rectal abscess | Dead |
| **7** | ATCL | Intermediate | Flu/Mel | MUD | Y / Y | LDH, sC5b9, Infection, aGVHD | 4 | 14 days | PR | 21 days | N/A | Alive |
| **12** | AML | Intermediate | Flu/Mel | MUD | Y / N | LDH, sC5b9, Infection, EOD | 3 | 20 days | CR | 9 days | N/A | Dead |
| **18** | MDS | Very-high | Flu/BCNU/Mel | MUD | Y / Y | LDH, sC5b9, Infection, aGVHD | 4 | 7 days | NR | N/A | Borderline (1,3)-beta-D-glucan elevation | Dead |
| **21** | ALL | Intermediate | Cy/TBI | Haplo | Y / N | LDH, EOD | 2 | <72 hrs | CR | 16 days | N/A | Alive |
| **49** | HL | Low | Flu/Mel | MRD | Y / Y | LDH, sC5b9, EOD | 6 | 6 days | CR | 8 days | N/A | Alive |
| **96** | ALL | High | Cy/TBI | Haplo | Y / Y | LDH, rUPCR, sC5b9, Infection | 4 | 6 days | NR | N/A | N/A | Dead |
| **98** | ALL | Intermediate | Flu/Mel | MRD | Y / N | LDH, rUPCR, sC5b9, aGVHD, EOD | 12 | >30 days | PR | 37 days | N/A | Dead |
| **101** | AML | High | Flu/Mel | MUD | Y / N | LDH, sC5b9, EOD | 4 | <72 hrs | CR | 17 days | Invasive candidemia | Dead |
| **145** | AML | High | Bu/Cy | MRD | Y / Y | LDH, aGVHD, Infection | 2 | 6 days | CR | 14 days | N/A | Dead |

Abbreviations – aGVHD: acute graft versus host disease, ALL: acute lymphoblastic leukemia, AML: acute myeloid leukemia, ATCL: angioimmunoblastic T-cell lymphoma, CNI: calcineurin inhibitor, CR: complete response, Cy: cyclophosphamide, DRI: disease risk index, Flu: fludarabine, HL: Hodgkin’s lymphoma, Mel: melphalan, MRD: matched-related donor, mTORi: mTOR inhibitor, MUD: matched-unrelated donor, N: no, NR: no response, PR: partial response, PRES: Posterior Reversible Encephalopathy Syndrome, TA-TMA: transplant-associated thrombotic microangiopathy, TBI: total body irradiation, Y: yes

** Armand P, et al. Blood. 2014 Jun 5;123(23):3664-71.*

*α Schoettler ML, et al. Transplant Cell Ther. 2023;29(3):151-163*

*Ɛ Transition to hospice 2 days after eculizumab initiation; response not assessable*
